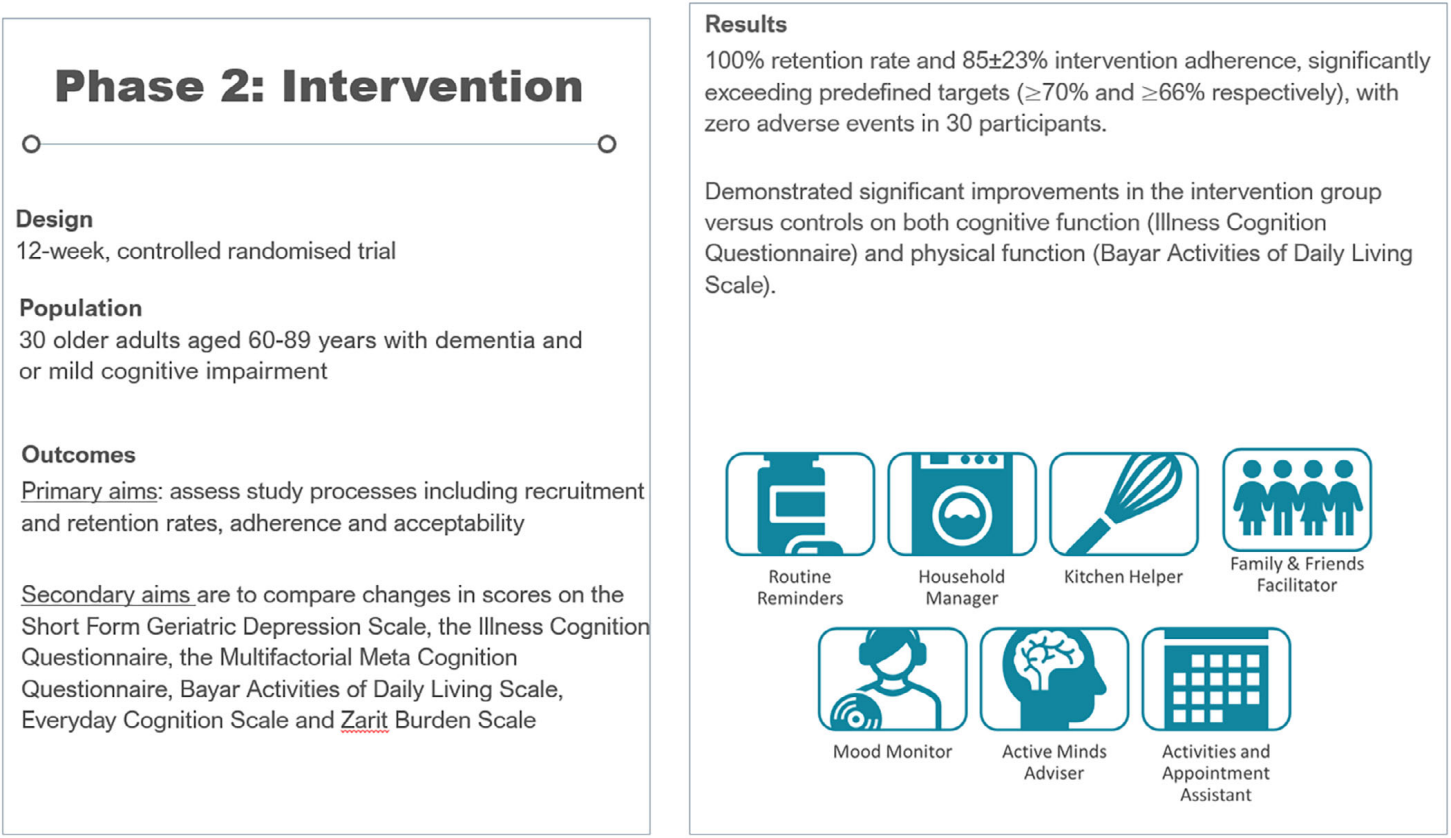# Cognitive Rehabilitation Programs for Older Adults with dementia using Digital Voice Assistants

**DOI:** 10.1002/alz70858_097936

**Published:** 2025-12-24

**Authors:** Paul S Jansons, Helen Macpherson, Michele L Callisaya, David S Scott, Tracy S Yiannis

**Affiliations:** ^1^ Deakin University, Melbourne, VIC, Australia; ^2^ Monash University, Melbourne, VIC, Australia; ^3^ National Centre for Healthy Ageing, Melbourne, VIC, Australia; ^4^ Peninsula Clinical School, Central Clinical School, Monash University, Melbourne, VIC, Australia; ^5^ Menzies Institute for Medical Research, University of Tasmania, Hobart, TAS, Australia

## Abstract

**Background:**

High healthcare costs and the poor outcomes associated with dementia have led to the application of new technologies to support management of people with dementia (PwD) in the community. To date, technology‐based support for post diagnostic care for PwD has primarily focused on safety monitoring and memory aids, and has largely ignored the need to support PwD to better self‐manage their condition. We recently developed and co‐designed an innovative digital voice assistant program (Dementia Australia Research Foundation ‐ Project Grant) that delivers personalised two way conversational post‐diagnostic care at home. It utilises natural language processing to interpret speech across all languages circumventing barriers using web‐based, touch screens or other digital input applications for PwD.

**Method:**

We conducted a 12‐week co‐designed feasibility and pilot randomised controlled trial of a digital voice assistant‐delivered personalised rehabilitative program in men and women with early dementia. We recruited 30 community‐based adults aged 60‐85 years and their carers residing anywhere in Australia with a recent (≤6 months) clinical diagnosis of early dementia (any type). Using a person‐centred approach to care, participants and carers consulted with a clinical psychologist to develop personally meaningful goals, in relation to improving cognition, function and/or activities of daily living delivered by our digital voice assistant program.

**Result:**

(*N* = 30/30) have shown mean±SD adherence to personalised, digital voice assistant program was 85±23% with no intervention‐related adverse events. System usability was rated above average (80.4±16.9 out of 100). Compared with controls, the intervention group significantly improved the Illness Cognition Questionnaire and the Bayar Activities of Daily Living Scale.

**Conclusion:**

A home‐based rehabilitative intervention delivered and monitored by health professionals using digital voice assistants was feasible for improving memory, cognition and activities of daily living in older adults with dementia. Future large‐scale, longer‐term studies are warranted to explore the clinical‐ and cost effectiveness of this digital health approach to supporting self‐management of dementia in older adults.